# Deep clinical and biological phenotyping of the preterm birth and small for gestational age syndromes: The INTERBIO-21 ^st ^Newborn Case-Control Study protocol

**DOI:** 10.12688/gatesopenres.12869.2

**Published:** 2019-02-05

**Authors:** Stephen H. Kennedy, Cesar G. Victora, Rachel Craik, Stephen Ash, Fernando C. Barros, Hellen C. Barsosio, James A. Berkley, Maria Carvalho, Michelle Fernandes, Leila Cheikh Ismail, Ann Lambert, Cecilia M. Lindgren, Rose McGready, Shama Munim, Christoffer Nellåker, Julia A. Noble, Shane A. Norris, Francois Nosten, Eric O. Ohuma, Aris T. Papageorghiou, Alan Stein, William Stones, Chrystelle O.O. Tshivuila-Matala, Eleonora Staines Urias, Manu Vatish, Katharina Wulff, Ghulam Zainab, Krina T. Zondervan, Ricardo Uauy, Zulfiqar A. Bhutta, José Villar

**Affiliations:** 1 Nuffield Department of Women's & Reproductive Health, University of Oxford, Oxford, UK; 2 Oxford Maternal & Perinatal Health Institute, Green Templeton College, Oxford, UK; 3 Programa de Pós-Graduação em Epidemiologia, Universidade Federal de Pelotas, Pelotas, RS, Brazil; 4 Oxford Ludwig Institute, University of Oxford, Oxford, UK; 5 Programa de Pós-Graduação em Saúde e Comportamento, Universidade Federal de Pelotas, Pelotas, RS, Brazil; 6 KEMRI-Coast Centre for Geographical Medicine and Research, University of Oxford, Kilifi, Kenya; 7 Centre for Tropical Medicine and Global Health, University of Oxford, Oxford, UK; 8 Faculty of Health Sciences, Aga Khan University, Nairobi, Kenya; 9 Faculty of Medicine, Department of Paediatrics, University of Southampton, Southampton, UK; 10 Department of Clinical Nutrition and Dietetics, College of Health Sciences, University of Sharjah, Sharjah, United Arab Emirates; 11 Big Data Institute, Li Ka Shing Centre for Health Information and Discovery, University of Oxford, Oxford, UK; 12 Shoklo Malaria Research Unit, Mahidol-Oxford Tropical Medicine Research Unit, Faculty of Tropical Medicine, Mahidol University, Mae Sot, Thailand; 13 Department of Obstetrics and Gynaecology, Division of Women and Child Health, Aga Khan University, Karachi, Pakistan; 14 Department of Engineering Science, University of Oxford, Oxford, UK; 15 SAMRC Developmental Pathways For Health Research Unit, Department of Paediatrics & Child Health, University of the Witwatersrand, Johannesburg, South Africa; 16 Centre for Statistics in Medicine, Botnar Research Centre, University of Oxford, Oxford, UK; 17 Center for Global Child Health, Hospital for Sick Children, Toronto, Canada; 18 Department of Psychiatry, University of Oxford, Oxford, UK; 19 Departments of Public Health and Obstetrics & Gynaecology, Malawi College of Medicine, Blantyre, Malawi; 20 Health, Nutrition & Population Global Practice, World Bank Group, Washington, DC, USA; 21 Nuffield Department of Clinical Neurosciences, University of Oxford, Oxford, UK; 22 Wellcome Trust Centre for Human Genetics, University of Oxford, Oxford, UK; 23 Division of Paediatrics, Pontifical Universidad Catolica de Chile, Santiago, Chile; 24 Department of Nutrition and Public Health Interventions Research, London School of Hygiene and Tropical Medicine, London, UK; 25 Department of Paediatrics and Child Health, Aga Khan University, Karachi, Pakistan

**Keywords:** preterm birth, SGA, fetal growth, INTERGROWTH-21st, INTERBIO-21st

## Abstract

**Background:** INTERBIO-21 ^st^ is Phase II of the INTERGROWTH-21 ^st^ Project, the population-based, research initiative involving nearly 70,000 mothers and babies worldwide coordinated by Oxford University and performed by a multidisciplinary network of more than 400 healthcare professionals and scientists from 35 institutions in 21 countries worldwide. Phase I, conducted 2008-2015, consisted of nine complementary studies designed to describe optimal human growth and neurodevelopment, based conceptually on the WHO prescriptive approach. The studies generated a set of international standards for monitoring growth and neurodevelopment, which complement the existing WHO Child Growth Standards. Phase II aims to improve the functional classification of the highly heterogenous preterm birth and fetal growth restriction syndromes through a better understanding of how environmental exposures, clinical conditions and nutrition influence patterns of human growth from conception to childhood, as well as specific neurodevelopmental domains and associated behaviors at 2 years of age.

**Methods:** In the INTERBIO-21 ^st^ Newborn Case-Control Study, a major component of Phase II, our objective is to investigate the mechanisms potentially responsible for preterm birth and small for gestational age and their interactions, using deep phenotyping of clinical, growth and epidemiological data and associated nutritional, biochemical, omic and histological profiles. Here we describe the study sites, population characteristics, study design, methodology and standardization procedures for the collection of longitudinal clinical data and biological samples (maternal blood, umbilical cord blood, placental tissue, maternal feces and infant buccal swabs) for the study that was conducted between 2012 and 2018 in Brazil, Kenya, Pakistan, South Africa, Thailand and the UK.

**Discussion:** Our study provides a unique resource for the planned analyses given the range of potentially disadvantageous exposures (including poor nutrition, pregnancy complications and infections) in geographically diverse populations worldwide. The study should enhance current medical knowledge and provide new insights into environmental influences on human growth and neurodevelopment.

## Introduction

The International Fetal and Newborn Growth Consortium for the 21 ^st^ Century (INTERGROWTH-21 ^st^) Project is a large, multicenter, population-based, research initiative, coordinated by the University of Oxford and being carried out by a multidisciplinary network of more than 400 healthcare professionals and scientists from 35 institutions in 21 countries worldwide. The project, involving nearly 70,000 mothers and babies, was established to assess human growth, neurodevelopment and associated behaviors from early pregnancy to 2 years of age under i) healthy conditions and ii) various sub-optimal conditions (e.g. maternal infections, malnutrition and pregnancy complications) and other risk factors for adverse outcomes. 

The Project’s overall mission was guided by a comprehensive series of conceptual papers ^1– 4^, systematic reviews ^5– 10^, epidemiological studies ^11, 12^ and evidence-based tools for providing continuity of clinical care ^4, 13^. The insights gained supported the project’s guiding principle: namely that the main negative perinatal outcomes—fetal death, preterm birth and fetal growth restriction (FGR)—are complex, inter-related syndromes that require targeted interventions focused on etiological factors.

Phase I of the INTERGROWTH-21 ^st^ Project, conducted between 2008 and 2015, consisted of nine complementary studies designed to describe optimal human growth and neurodevelopment, based conceptually on the World Health Organization (WHO) prescriptive approach ^14^. Across eight urban areas worldwide, which were geographically delimited to ensure the study was population-based ^15^, we enrolled a large cohort of healthy pregnant women before 14 ^+0^ weeks’ gestation. The specific aim was to monitor their babies prospectively until 2 years of age so as to generate international standards for: i) estimating gestational age in early and late pregnancy ^16, 17^; ii) monitoring symphysis-fundal height ^18^ and maternal weight gain ^19^; iii) measuring fetal size and estimated fetal weight with ultrasound to monitor fetal growth ^20, 21^; and iv) assessing newborn size for gestational age ^22, 23^, newborn body composition ^24^ and the postnatal growth of preterm infants ^25^. Up to 2 years of age, children included in this cohort remained healthy with adequate growth and motor development, supporting its appropriateness for the construction of international standards ^26^.

In addition, the sequence and timing of attainment of key neurodevelopmental milestones and associated behaviours among these children at 2 years of age were assessed using a tool specifically constructed for the Project ^27^, for implementation by non-specialists across international settings ^28^. We have demonstrated that developmental patterns were similar across these geographically diverse populations when health, nutritional and environmental risks were very low ^29^.

Considerable global impact has been achieved in the 3 years since the initial results of the INTERGROWTH-21 ^st^ Project were published. For example, the growth standards, which perfectly complement the existing WHO Child Growth Standards ^30^, have been adopted by WHO ^31^; the Centers for Disease Control and Prevention (CDC) ^32^; the Ministries of Health of Brazil, Haiti, New Zealand and Sri Lanka; the National Pediatric Society of Argentina and the Italian Society of Neonatology. Moreover, the INTERGROWTH-21 ^st^ set of clinical tools, freely available at https://intergrowth21.tghn.org, have been downloaded 111,719 times by users across the world (updated 29 August 2018), and more than 10,000 health care professionals have been trained using the INTERGROWTH-21 ^st^ e-learning modules ( https://globalhealthtrainingcentre.tghn.org). In addition, the INTERGROWTH-21 ^st^ Neurodevelopment Package has been implemented in over 6500 children in 14 countries, and more than 100 health care professionals have been trained in its use. The operation manuals and protocols for the Package are freely available at www.inter-nda.com.

More recently, the Child Health Epidemiology Reference Group has selected the INTERGROWTH-21 ^st^ Newborn Size at Birth Standards for gestational age/sex ^22^ as the most reliable tool for estimating the prevalence of small for gestational age (SGA) in low- to middle-income countries (LMICs) worldwide. They reported that 23.3 million infants were born SGA in 2012; among these, 11.2 million were term and not low birth weight (LBW, ≥2500 g), 10.7 million were term and LBW (<2500 g) and 1.5 million were preterm ^33^.

The cohort studied in Phase I was selected because the participants had, both at the population and individual level, the recommended health, nutritional and socio-economic status required to construct international standards, i.e. these were generally healthy, well-nourished, well-educated mothers living freely in environments with minimal constraints on fetal growth, whose antenatal care was evidence-based and standardized. Interestingly, the infants in this cohort from LMICs are predicted as adults to be approximately 8 cm taller than the mean height of their parents, assuming that their health, nutritional and socio-economic conditions remain adequate ^26^.

These results, therefore, confirm a pattern and magnitude of transgenerational ‘washout’ ^34^, that was also seen in the populations contributing to the WHO Child Growth Standards ^35^. This effect on skeletal growth, which can seemingly occur in one generation, almost certainly represents a response to environmental changes such as improvements in nutrition and health care. However, the mechanisms responsible, which may include modifications in gene expression not linked to DNA sequence changes, are still to be determined ^36^.

Phase II of the INTERGROWTH-21 ^st^ Project (The INTERBIO-21 ^st^ Study) aims to improve the functional classification of our previously evaluated, preterm birth and FGR syndromes ^11, 12^ through a better understanding of how environmental exposures, clinical conditions and nutrition influence patterns of human growth from conception to childhood. We expect to extend this concept to specific neurodevelopmental domains and associated behaviors at 2 years of age at both the individual and population level.

The INTERBIO-21 ^st^ Study’s hypotheses are driven by the concept that improvements in the phenotypic characterization of these complex syndromes through the integration of clinical and laboratory data might facilitate the development of targeted screening and preventive strategies, as well as interventions in the periconceptual period, pregnancy and infancy. In addition, we believe this approach will reveal valuable insights into the role of biological factors in high-risk populations. The need is urgent given the limited effect of previous efforts, e.g. interventions delivered non-specifically to high-risk populations to prevent preterm birth and FGR as if these were single clinical entities ^37^.

In the INTERBIO-21 ^st^ Newborn Case-Control Study, a major component of Phase II, our objective is to investigate the mechanisms potentially responsible for preterm birth and SGA and their interactions, using deep phenotyping of clinical, growth and epidemiological data and associated nutritional, biochemical, omic and histological profiles. Here we describe the study sites, population characteristics, study design, methodology and standardization procedures for the collection of longitudinal clinical data and biological samples from fetuses, newborns and young children who were exposed to a variety of potentially disadvantageous intrauterine environments (including poor nutrition, pregnancy complications and infections) in geographically diverse populations worldwide.

## Study design principles

The study design principles were included in an original version of this protocol, which has been available on the study website mainly for our collaborators’ use ( https://www.interbio21.org.uk).

Selection of the appropriate control sample in case-control studies is one of the most complex issues in epidemiological design, and also one in which apparent common sense may prove to be wrong, in particular the notion that controls had to be “healthy” or “normal” as opposed to only free of the disease being studied, i.e. non-cases ^38^. 

There are currently two key concerns regarding selection of “controls”. First, controls should represent the population from which the cases were selected. This will ensure internal validity of the study by avoiding selection bias. It will provide a more realistic measure of the magnitude of the association. It is not required that controls should be healthy in all respects because in the population from which the cases came there will be ‘unhealthy’ subjects with clinical or subclinical problems or pathologies.

Second, control selection should be driven by the epidemiological measure of effect that one wishes to estimate. In etiological research, the most appropriate measure of effect is the incidence density ratio (IDR), or rate ratio, which is equal to the ratio between the incidence rates in the exposed and unexposed groups. Nevertheless, it is not always possible to estimate the IDR directly in case-control designs if the study population is not followed up over time.

The two most common designs differ according to the type of controls selected. These are the case-non-case design and the case-base design. In the former, non-case controls include newborns ≥38 ^+0^ weeks’ gestation, regardless of whether or not they are SGA. As there are many more potential controls than cases, controls were sampled to improve the efficiency of the study, and to avoid carrying out expensive tests on all non-cases. The case-non-case design is easy to explain and provides an estimate of the odds ratios associated with specific exposures, which is a good estimate of the IDR when rates of delivery <38 ^+0^ weeks’ gestation are relatively low, but will overestimate the IDR if the delivery rate <38 ^+0^ weeks is high. Logistic regression is the method for analyzing case-non-case designs when prevalence is, say, less than 10%, and Poisson regression with robust variance when prevalence is higher ^39^.

In the case-base design, controls are sampled from all pregnant women, including those who delivered <38 ^+0^ weeks’ gestation. Such women are, therefore, included as both cases (all women delivering at <38 ^+0^ weeks’ gestation) and controls (rather a sample of these women, using the same sampling fraction as for women delivering ≥38 ^+0^ weeks). The case-base design estimates the prevalence ratio—it is important to remember that prevalence is obtained by dividing subjects with a given characteristic (for example, birth at <38 ^+0^ weeks’ gestation) by the whole population, which includes all births. This justifies the inclusion of some women with births at <38 ^+0^ weeks’ gestation in the control group as well. Prevalence ratios obtained from a case-base design tend to overestimate the IDR for births at <38 ^+0^ weeks’ gestation, particularly when the rate of delivery at <38 ^+0^ weeks’ gestation is high. By collecting data on the four subgroups of births (A, B, C and D, see Table 1), it is possible to use weighting to reproduce a case-base analysis. Analyses of case-base designs may be carried out using Poisson regression with robust variance.

**Table 1.** Groups included in the INTERBIO-21 ^st^ Newborn Case-Control Study.

Set	Babies born <38 ^+0^ weeks’ gestation	Babies born SGA	Description	Number to be included in case-control study
A	Yes	No	Non-SGA babies born <38 ^+0^ weeks’ gestation	A (all)
B	No	Yes	SGA babies born ≥38 ^+0^ weeks’ gestation	B (all)
C	Yes	Yes	SGA babies born <38 ^+0^ weeks’ gestation	C (all)
D	No	No	Non-SGA babies born ≥38 ^+0^ weeks’ gestation	Sample = A+B+C

SGA, small for gestational age.

For SGA, the same principles discussed above apply with a few modifications. For both case-non-case and case-base designs, cases include SGA newborns, defined as birth weight for gestational age/sex of the INTERGROWTH-21 ^st^ Newborn Size Standards ^22^. In the case-non-case design, controls would be a sample of all newborns who do not present SGA at birth. The measure of effect would be the odds ratio, which overestimates the IDR and the prevalence ratio when SGA prevalence exceeds 10%. In the case-base design, SGA at birth is a point prevalence measure, more specifically the proportion of all babies born with low weight for their gestational age/sex. For example, 12% of all newborns in a population may present SGA (note that the denominator of the prevalence measure includes births with and without SGA). The case-base design directly estimates the prevalence ratio, because the control group includes a sample of all births, regardless of their gestational age at delivery or SGA status. It may be argued that for SGA the prevalence ratio is a better measure than the IDR, in particular given how hard it is to define the precise incidence and timing of SGA onset.

By collecting data on the four subgroups of births (A, B, C and D; see Table 1), it is possible to carry out both case-non-case and case-base analyses in the INTERBIO-21 ^st^ Newborn Case-Control Study. Initially, we propose that controls should be selected from non-cases and that the primary analyses should entail case-non-case comparisons. We also plan to carry out analyses using a case-base approach, by using statistical weighting to correct for the over-sampling of infants born <38 ^+0^ weeks’ gestation and those that are SGA, thereby reproducing the whole population of births. Further details are provided below.

## Methods

### Study sites

The INTERBIO-21 ^st^ Study was conducted between February 2012 and June 2018 at seven sites: Pelotas (Brazil), Kilifi (Kenya), Nairobi (Kenya), Karachi (Pakistan), Soweto (South Africa), Mae Sot (Thailand) and Oxford (UK), of which two (Kilifi and Mae Sot) were rural and the others urban. The sites in Pelotas, Nairobi and Oxford also participated in Phase I of the INTERGROWTH-21 ^st^ Project ^40– 42^.

*Pelotas (Brazil)*: The middle-income city of Pelotas, in the southernmost region of the country where the richer Brazilian states are located, was also the Latin American site for Phase I ^40^. Pelotas is the third most populous city in the state of Rio Grande do Sul, with 350,000 inhabitants (92% living in urban areas) and 4,000 births per year. More than 99% of these births take place in the city’s four maternity hospitals. In 2007, Pelotas had a per capita gross domestic product (GDP) of R$8248 (US$4933). A total of 47% of women in Pelotas receive >9 years of formal education, with 21% receiving more than 12 years. Data from the Pelotas 2004 birth cohort study indicate that the LBW and FGR rates are 10% and 12%, respectively, and that the mean birth weight of new-borns is 3150 g ^43^. The city has an Epidemiology Research Centre based at the Federal University of Pelotas, which has been conducting epidemiological research on maternal and child health nutrition for more than 30 years and is also a WHO Collaborating Centre in the field of nutrition. The same research team also participated in the WHO Multicentre Growth Reference Study (MGRS), which generated the WHO Child Growth Standards ^44^. 

*Kilifi (Kenya)*: Kilifi County Hospital (KCH) is located in a rural, malaria endemic, coastal area, 55 km north of Mombasa, which is the second poorest county in Kenya. The hospital, has a catchment area of approximately 280,000 people with 3,000 births per year. The antenatal HIV prevalence is 7.9% ^45^. KCH hosts the Kenyan Medical Research Institute (KEMRI)/Wellcome Trust Research Programme, a partnership with the University of Oxford that, since 1989, has pioneered work on laboratory-based, epidemiological and clinical research. In the late 1990s, an antenatal ultrasound service was established facilitating pregnancy-related research. The research program has a computerized, Health and Demographic Surveillance System (KHDSS) that catalogues a sub-population of people living in KCH’s predominant catchment area ^46^. Each individual is given a personal identification number and births, deaths, in- and out-migration are recorded at 4-monthly household visits. The KHDSS provides a means to encourage attendance at antenatal clinics, particularly for booking early in pregnancy. 

*Nairobi (Kenya)*: The relatively wealthy Parklands suburb of Nairobi, Kenya, was the sub-Saharan site for Phase I ^41^. Almost all births (>4,000) in this geographically delimited urban area, which mostly houses affluent Kenyan families, take place in three hospitals, the largest of which, The Aga Khan University Hospital (AKUH), participated in the study. AKUH is a private, not-for-profit, tertiary care institution, which is accessed predominantly by the middle and high socio‐economic sectors. These women are able to access the hospital services either through medical insurance cover or direct payment; thus, the pregnant population served is at relatively low risk of FGR. In 2008, the mean birth weight was 3101 g; the low birth weight and perinatal mortality rates were 11% and 1.7%, respectively. Nairobi is a non-endemic malaria area but HIV remains a significant problem in the city with a prevalence of 10% in the female population although much lower at 1% among women attending this hospital for antenatal care. Thanks to wide access to drugs and other aspects of care, during the period of this study almost all women living with HIV or newly diagnosed during antenatal screening had commenced antiretroviral therapy.

*Karachi (Pakistan)*: The Aga Khan University Hospital (AKUH) is a philanthropic, not-for-profit teaching institution, which caters to a range of socio-economic groups through an effective patient welfare program. The hospital is the most advanced private-sector tertiary care institution in Karachi, the largest city in Pakistan with an estimated population of about 20 million people. The AKUH is also affiliated with four secondary care hospitals (combined total approximately 15,000 births per year) from which high-risk cases are referred as needed: three in Karachi and one about 100 miles away. The main AKUH has 5,800 births per year, a third of which are high-risk. The per capita income in Karachi is approximately $1,427. At AKUH, the first antenatal visit is usually in the first trimester, at which time a dating scan is performed ^47^. Nuchal translucency screening is offered to high-risk women and a fetal anomaly scan is offered between 19–23 weeks’ gestation.

*Soweto (South Africa)*: Chris Hani Baragwanath Academic Hospital (CHBAH), which is attached to the University of the Witwatersrand, Johannesburg, South Africa, is the only government hospital serving two sub-districts in southwestern Johannesburg, which includes the Greater Soweto area, with a population of approximately 1.3 million. CHBAH provides district hospital referral services for seven community health centers in Greater Soweto, in which midwives conduct low-risk births; approximately 5% of births are tertiary referrals from other districts and provinces. The hospital has approximately 17,000 births per year, 75% of which are medium-to-high risk; this represents 53% of all births in Greater Soweto ^48^. The HIV prevalence is 29% ^49^. The population is urbanized, indigenous African, and working-class. The average annual income is substantially less than the national average, at approximately US$4,000. CHBAH is linked to the Soweto First 1000 Days Study (S1000) at the MRC/Wits Departmental Pathways for Health Research Unit (DPHRU) ^50^.

*Mae Sot (Thailand)*: The Shoklo Malaria Research Unit (SMRU), located on the Thailand-Myanmar border, is a field station of the Mahidol-Oxford Research Unit (MORU) of Mahidol University, Bangkok. SMRU was established in 1986 as a research centre for studying the epidemiology, prevention and treatment of resistant malaria (including in pregnancy) among the 130,000 people living in refugee camps along the border. As there is no safe and effective drug for preventing *Plasmodium falciparum* and *Plasmodium vivax* in pregnancy, women are encouraged to attend the antenatal clinic at SMRU every 2 weeks from as early as possible in the first trimester, and they are systematically screened for malaria at each antenatal consultation and treated if positive ^51^. In addition, since 2008, local health workers with limited education have been trained at SMRU to take fetal growth measurements with great accuracy ^52^. Three of its field sites, with a total of approximately 2,100 births per year, participated in the study. In 2008, in newborns >28 weeks’ gestation, the mean birth weight was 2908g; the LBW and perinatal mortality rates were 15.6% and 3.4%, respectively.

Of the sites, two (Wang Pha and Mawker Thai) are clinics for migrants; the third, the Maela camp, is the largest refugee camp along the border. Both populations remain marginalized: the educational level is low and most of available income is obtained through intermittent work paid below the minimum wage and some women in the camp receive a supplement of refugee food rations. In 2012–13, HIV prevalence was low in refugees and migrants: 0.27% and 0.61%, respectively ^53^.

*Oxford (UK)*: The John Radcliffe Hospital, Oxford was one of the two sites in Europe that participated in Phase I ^42^. Oxfordshire has a population of more than 650,000 people, which includes a large proportion of young, middle‐class, well‐educated, professional families. A total of 37% of the Oxfordshire population hold a university degree, 16% higher than the national average. The hospital covers approximately 75% of more than 8,000 pregnancies that occur annually in this county. The general pregnant population served is at low risk of FGR. In 2008, the mean birth weight was 3334 g; the LBW and perinatal mortality rates were 6% and 0.5%, respectively. In addition, 99% of mothers delivering in the unit have completed secondary school or university level education. The hospital also houses the University of Oxford’s Nuffield Department of Women’s & Reproductive Health, which is where the INTERGROWTH‐21 ^st^ Project Coordinating Unit is located.

These sites contributed cases and controls to the INTERBIO-21 ^st^ Newborn Case-Control Study, selected using the definitions described below.

### Case-control definitions

The INTERBIO-21 ^st^ Newborn Case-Control Study consisted of two components evaluating pregnancy characteristics, birth outcomes, neurodevelopment and biological markers associated with the preterm birth and SGA syndromes. 

The first component aimed to compare preterm phenotypic cases to term newborn controls.

*Preterm cases* were singleton, naturally conceived babies, live-born at 23 ^+0^ to 37 ^+6^ weeks’ gestation ^2, 3^, whose mothers were ≥18 years of age and resided in the hospital’s catchment area (to avoid recruiting women referred for tertiary care from another geographical region), and whose gestational age was estimated by ultrasound measurement of either crown-rump length <14 ^+0^ weeks’ gestation or head circumference <24 ^+0^ weeks’ gestation ^54^. For the planned analyses, we will stratify the cases by gestational age (defined *a priori)* into those born <37 ^+0^ weeks, and those born ≥37 ^+0^ but <38 ^+0^ weeks’ gestation, and according to the previously described phenotypes to explore interaction effects ^11^. Cases include groups A and C ( Table 1).

For the case/non-case analyses, *controls for preterm cases* were singleton, naturally conceived babies, live-born at 38 ^+0^ to 41 ^+6^ weeks’ gestation, and appropriately grown for gestational age (AGA), i.e. with a birth weight for gestational age/sex ≥10 ^th^ centile of the INTERGROWTH-21 ^st^ Newborn Size Standards ^22^, whose mothers were ≥18 years of age and resided in the hospital’s catchment area, and whose gestational age was estimated by ultrasound measurement of either crown-rump length <14 ^+0^ weeks’ gestation or head circumference <24 ^+0^ weeks’ gestation ^54^. These controls include groups D and B. For group B, the sample will be down-weighted to represent their actual occurrence in the population.

A cut-off of 37 ^+6^ weeks instead of 36 ^+6^ weeks’ gestation was used to define a preterm case because of the evidence of a small but nevertheless increased risk of respiratory and other adverse neonatal outcomes (including mechanical ventilation, sepsis, hypoglycemia, NICU admission, and hospitalization for 5 days or more) in those ‘term’ babies born between 37 ^+0^ and 37 ^+6^ weeks’ gestation ^55^.

The second component aimed to compare SGA phenotypic cases to term newborn controls.

*SGA cases* were singleton, naturally conceived babies, live-born at 23 ^+0^ to 41 ^+6^ weeks’ gestation, with a birth weight for gestational age/sex <10 ^th^ centile of the INTERGROWTH-21 ^st^ Newborn Size Standards ^22^, whose mothers were ≥18 years of age and resided in the hospital’s catchment area, and whose gestational age was estimated by ultrasound measurement of either crown-rump length <14 ^+0^ weeks’ gestation or head circumference <24 ^+0^ weeks’ gestation ^54^. Cases include groups B and C ( Table 1).

For the case/non-case analyses, *controls for SGA cases* were singleton, naturally conceived babies, live-born at 38 ^+0^ to 41 ^+6^ weeks’ gestation, and AGA, i.e. with a birth weight for gestational age/sex ≥10 ^th^ centile of the INTERGROWTH-21 ^st^ Newborn Size Standards ^22^, whose mothers were ≥18 years of age and resided in the hospital’s catchment area, and whose gestational age was estimated by ultrasound measurement of either crown-rump length <14 ^+0^ weeks’ gestation or head circumference <24 ^+0^ weeks’ gestation ^54^. These controls include groups D and A. For group A, the sample will be down-weighted to represent their actual occurrence in the population.

For the second analytical approach—the case-base analyses—controls will include newborns from all four groups. For groups A, B and C, the samples will be down-weighted to represent their actual occurrence in the population.

Cases were recruited consecutively and one newborn control was recruited immediately after each preterm case was recruited; similarly, another newborn control was recruited immediately after each SGA case was recruited. Both sets of newborn controls will be pooled to create a control group for use in the comparative analyses with both preterm and SGA cases separately (as well as those cases born preterm and SGA), resulting in two newborn controls per case and a considerable increase in statistical power.

At all sites, trained, dedicated research staff screened all women presenting for delivery on a daily basis using a tablet (iPad, Apple, USA)-based interface with the data management system ( https://doi.org/10.5281/zenodo.1442668 ^56^). The software (available on request), that was specially written for the study, selected the correct proportion of preterm and SGA cases and corresponding controls according to birth weight and gestational age. Thus, each newborn recruited fell into one of the four groups shown in Table 1.

The software selected a higher proportion of newborns with earlier gestational ages (for preterm cases) and lower birth weights, i.e. <3 ^rd^ centile (for SGA cases), using the sampling fractions shown in Table 2 so as to avoid recruiting excessive numbers just below the cut-offs that represent the majority of SGA and preterm newborns, i.e. moderate SGA and late preterm newborns. Oversampling cases at the lower end of the gestational age and birth weight distributions was important to have a large enough sample size to study the highest risk sub-groups; it was also expected to increase the statistical power of the study by producing a higher proportion of exposures and adverse neonatal outcomes.

**Table 2.** Sampling fractions for recruitment in the INTERBIO-21 ^st^ Newborn Case-Control Study.

GA, or BW for GA centile (C)	To be recruited, %	Case/Control
GA <36 ^+0^ weeks (up to and including 35 ^+6^)	100%	Preterm case
GA 36 ^+0^ to 36 ^+6^ weeks	50% ➔ 100% *	Preterm case
GA 37 ^+0^ to 37 ^+6^ weeks	5% ➔ 20% *	Preterm case
BW/GA <C3 ^rd^ ➔ <C5 ^th #^	100%	SGA case
BW/GA C3 ^rd^ - C9.9 ^th^ ➔ C5 ^th^ – C9.9 ^th #^	50%	SGA case
GA 38 ^+0^ to 41 ^+6^ weeks and BW/GA ≥ C10 ^th^	Will vary according to the number of cases recruited	Potential controls to be sampled immediately after each case

GA, gestational age; BW, birth weight. *Change occurred in November 2012. ^#^Change occurred in November 2013.

Slight changes in the sampling fractions (see arrows in Table 2) were recommended by the study’s epidemiological advisors and introduced for preterm cases in November 2012 and for SGA cases in November 2013 to reach the recruitment rates initially planned. These changes were anticipated because the actual recruitment rate of cases was difficult to predict. The adjustments were facilitated by the tablet software.

We aimed to recruit at least 2,000 cases and 2,000 controls in total from the study sites. However, we recognised then that power calculations are a great challenge in any field-study of this magnitude and even more difficult when exploring risk factors with relatively unknown degrees of association and prevalence in such populations. The key issue is to reach a balance between logistical demands, including the need to maintain data quality in these populations, and power calculations especially for the planned genetic and epigenetic studies. In addition, when the study was designed in 2012, it was extremely difficult to provide reliable power calculations for epigenetic studies: the field was too new and very few relevant studies had been conducted. The compromise was to use experience gained from genome-wide association studies to facilitate sample size estimations. Thus, 1,500 cases and 1,500 controls (ratio 1:1) would be required, assuming a methylation proportion of 0.3 and 0.2 in cases and controls, respectively, to detect an odds ratio of 1.7 (population attributable fraction of 0.12) with a significance threshold alpha of 5.0 × 10 ^-7^ and 90% power. These calculations included a continuity correction allowing for normal approximation of the binomial distribution.

### Gestational age estimation by ultrasound

The methods used to estimate gestational age, as well as the training, standardization and quality control processes are described elsewhere ^57– 59^. In brief, crown-rump length measurements were taken <14 ^+0^ weeks’ gestation in a mid-sagittal view of the horizontal fetus in a neutral position, with an angle of insonation as close as possible to 90°. The image could not fill less than 30% of the monitor screen. The callipers were placed on the outer borders of the head and rump, and gestational age was estimated using the INTERGROWTH-21 ^st^ standards for pregnancy dating ^16^.

Head measurements were taken <24 ^+0^ weeks’ gestation in an axial view at the level of the thalami, with an angle of insonation as close as possible to 90° using the same ultrasound machine at each site (Philips HD-9, Philips Ultrasound, USA with curvilinear abdominal transducers C5-2, C6-3, V7-3). The head had to be oval in shape, symmetrical, centrally positioned, filling at least 30% of the monitor. The midline echo (representing the falx cerebri) had to be broken anteriorly, at one-third of its length, by the cavum septum pellucidum. The thalami had to be located symmetrically on either side of the midline. The head circumference was measured using the ellipse facility on the outer border of the skull, and gestational age was estimated using the INTERGROWTH-21 ^st^ standards for late pregnancy dating ^17^.

Femur length was measured using a longitudinal view of the fetal thigh closest to the probe and with the femur as close as possible to the horizontal plane. The angle of insonation of the ultrasound beam was approximately 90° with the full length of the bone visualised, unobscured by shadowing from adjacent bony parts and the femur had to fill at least 30% of the monitor screen. The intersection of the callipers was placed on the outer borders of the edges of the femoral diaphysis (outer to outer) ensuring clear femoral edges; ultrasound artefacts of the femoral edges such as the proximal “trochanter” or pointed femoral spurs were not included in the measurement (the detailed methodology and a graphical display of how the bone structures are localised are available at www.intergrowth21.org.uk). 

The ultrasonographers at each site were selected on the basis of their technical expertise, motivation, reliability and ability to speak the local language(s). Through rigorous training they gained theoretical knowledge and familiarity with the study protocol, operations manual, data collection and quality control measures. Centralized hands-on training and initial standardization were also conducted ^60^, and the Oxford-based Ultrasound Quality Control regularly carried out site-specific standardization to ensure proper use of the ultrasound equipment, calibration and adherence to the protocol. Quality control was maintained throughout the study by taking a random 10% sample of all ultrasound images and assessing their quality using a validated scoring system ^58^.

### Anthropometric measures

The anthropometric measurement protocols and quality control procedures were identical to those used in Phase I ^61, 62^. A team of anthropometrists was specially recruited, trained and standardized for the study; all training materials were based on the original WHO MGRS protocols ^44^. 

In brief, newborn anthropometric measures were ideally obtained in all neonates within 12 h of delivery (and no later than 24 h), using identical equipment at all sites: electronic scale for birth weight (Seca, Hamburg, Germany) and a specially designed Harpenden infantometer (Chasmors Ltd, London, UK) for recumbent length. The equipment was selected for accuracy, precision and robustness, as reported in previous studies ^44^, and calibrated twice weekly. Head circumference was measured using a metallic non-extendable tape (Chasmors Ltd, London, UK). All lead anthropometrists were trained centrally and, in turn, trained the local anthropometrists to measure newborns according to the study protocol. The Anthropometric Standardization Unit based in Oxford regularly monitored the performance of all the anthropometrists.

The quality control measures required anthropometrists at each study site to take and record all measurements independently and compare their values with the maximum allowable differences. They also checked the forms visually after each session to ensure appropriate remeasurements were performed when necessary ^62^.

### Neonatal outcomes

We will use an un-weighted composite outcome including at least one of the following conditions: neonatal death until hospital discharge of the newborn, stay in NICU for ≥7 days or other severe neonatal complications, such as intraventricular hemorrhage and necrotizing enterocolitis. We have used such a composite outcome (that, when appropriate, also included stillbirths) previously ^63, 64^; it requires limited standardization of clinical diagnoses across hospitals and is well accepted as a marker in large, international, population-based studies of newborns that are severely ill ^65, 66^. We believe this is a good proxy for adverse neonatal outcomes across countries.

### Biological sample processing and storage: INTERBIO-21 ^st^ Biorepository

Biological samples were collected from participants to establish a biorepository for a series of planned nutritional, biochemical, omic and histological studies. The protocols for processing and storing the samples are described briefly below. For each sample type, kits containing all the necessary supplies, including tubes pre-labelled with a unique aliquot identifier, were prepared by GAPPS (Global Alliance to Prevent Prematurity and Stillbirth, Seattle, USA) and supplied to each participating site.

Prior to sample collection beginning, the lead laboratory technician from each site was brought to Oxford for a centralized training session. Everyone was trained in collecting, processing and storing samples, as well as recording the associated data. Further training sessions were conducted on-site for the full laboratory teams every 6–12 months by the global laboratory lead to ensure adherence to the protocols and to retrain any staff if necessary.

*Maternal blood*: Maternal blood was collected at delivery and routinely processed within 12 h of collection. Plasma was divided into up to six 1-ml aliquots; the buffy coat was aspirated slowly using a circular motion and stored in a pre-labelled tube. After gentle inversion to ensure thorough mixing, the whole blood in the EDTA tubes was divided into 1.5 ml aliquots. After processing, all relevant information on the collection and processing of the plasma, buffy coat and whole blood specimens was recorded on the specially designed e-forms and the aliquots were stored at −80°C.

*Umbilical cord blood*: Cord blood was collected within 30 mins of delivery of the placenta (or whilst the placenta remained *in utero*). After gentle inversion to ensure thorough mixing, the whole blood in the EDTA tubes was divided into 1.5 ml aliquots before being stored at -80°C. After collection, the blood in the trace element tube was inverted to ensure thorough mixing and then left for 30 mins before processing to prevent the formation of a gel disc. It was then centrifuged for 10 mins at 1200 *g* to separate the plasma (top layer) and buffy coat (middle white layer), both of which were retained, from the bottom layer of red blood cells that was discarded. The plasma was stored in up to three 1 ml aliquots in Nalgene polypropylene tubes (Fisher Scientific, Leicestershire, UK); the buffy coat was aspirated slowly using a circular motion and also stored in a Nalgene polypropylene tube. These tubes were selected as they allow subsequent trace element analysis of the plasma sample. After processing, all relevant information on the collection and processing of the whole blood, plasma and buffy coat was recorded on e-forms and the aliquots were stored at -80°C.

*Placenta*: If processing within 1 h of delivery was possible, two placental tissue punches (~8 mm diameter x full placenta thickness) from the placental disc, avoiding the site of umbilical cord insertion and at least 3 cm from the edge of the placenta, were collected and placed in a tube preloaded with 3 ml RNAlater (Sarstedt, Nümbrecht, Germany) for future estimation of RNA. A 0.5 cm membrane strip, cut using scissors or a scalpel from the rupture site to the edge of the placental disc, was also placed in a tube preloaded with 3 ml RNAlater. These samples were stored at 4°C for a minimum of 24 h and a maximum of 4 weeks before freezing at −80°C. In an area of the placenta adjacent to the RNAlater sampling points, two tissue punches of similar size (1 cm diameter x full placenta thickness) and a small sample of membrane (1 cm wide) were frozen in liquid nitrogen or dry ice. A second membrane sample (1 cm wide) and sample of the placental disc were placed in formalin for future histology. These samples were stored at room temperature for 48–72 h after which the formalin was removed, the tissue washed with 70% ethanol and then transferred to a tube containing 4 ml 70% ethanol for long-term storage. 

If the samples could not be processed within 1 h, the placenta was stored at 2–8°C for later processing. Within 12 h of delivery, samples were collected for freezing and histology (but not RNA estimation); if sample processing was possible only at >12 but <24 h of delivery, samples were collected for histology only. 

Two photographs were taken of the placenta showing the whole placenta and umbilical cord with the ‘fetal’ and ‘maternal’ sides uppermost. A metric ruler was placed at right angles next to the tissue to indicate the size of the placenta. The weight of the placenta, trimmed of the cord and all its membranes, was recorded.

*Maternal feces*: A sample of maternal feces (approximately 5 g), if passed at delivery, was collected and stored at -80°C.

*Buccal swabs*: DNA was collected from the infants at 1 and 2 years of age using a buccal swab collection kit (MAWI DNA Technologies, CA 94545, USA). Up to four swabs were gently rubbed against the inside of the infant’s cheek and placed into a single vial of buffer. After collection, the swabs were discarded and the cloudy buffer containing the DNA was stored and transported at ambient temperature.

### Data management system

All clinical data were managed in a system very similar to the one used in Phase I of the INTERGROWTH-21 ^st^ Project ^67^. In brief, the data were initially collected on paper forms capturing information relating to ultrasound estimation of gestational age, pregnancy & delivery, and any fetal/neonatal abnormalities; these forms were securely stored. The data were then entered at the local level into an on-line data management system, based on the one developed specifically for the INTERGROWTH-21 ^st^ Project (MedSciNet, London, UK). This on-line system, which resides on a secure MedSciNet server, facilitated quality control, correction of errors or missing values, and the initiation of data analysis soon after completion of data collection. A review process within the system, which involved weekly queries to each site via Skype if necessary, ensured that all key data were complete. Blinded data from the ultrasound machines were transferred directly to the database in Oxford. 

All sample-related data were collected on an electronic form (e-form) and the data were uploaded onto a separate data management system (Sapphire, Labvantage Solutions Ltd, High Wycombe, UK) that was specifically modified for the study. This system, which resides on a secure University server, allows samples to be tracked from the time of collection through processing, storage at the study sites, and transport to the central storage facility in Oxford. Each participant was given a unique identifier number, which was used to link the clinical and sample databases. Individual aliquots of each sample type were also given a unique number.

All the electronically stored data were stripped of personal identifiers, which are held separately and securely on site. The anonymised databases are only accessible to designated personnel, including the Bill & Melinda Gates Foundation as part of a data sharing agreement. Users from each study site can only view their own data at present and a limited number of global administrators can see all the data on a secure server.

These systems provided the Data Management Unit in Oxford with a detailed daily record of patient enrolment and data entry, at both individual and institutional levels, to monitor progress. Corresponding actions, such as telephone calls, web conferences and site visits took place within a week of detecting a problem at a study site to ensure that appropriate corrective measures were taken.

## Ancillary studies

### INTERBIO-21 ^st^ Newborn Body Composition Study

Body composition was estimated within 96 h of birth at two study sites, Mae Sot and Oxford, using air displacement plethysmography (ADP) (PEA POD®, COSMED, Rome, Italy), which derives the proportion of fat mass (FM) and fat-free mass (FFM) by measuring body volume (air displacement) and weight. ADP has considerably improved our understanding of newborn body composition and the implications of feeding regimens especially for preterm and growth restricted newborns.

This non-invasive technique is rapid, reliable, robust to moderate levels of infant activity, and acceptable to parents ^68^. Using the same PEA POD methodology, we have previously reported normative data at term following the prescriptive approach, plus differential FM, body fat percentage and FFM patterns for babies born preterm (34 ^+0^ to 36 ^+6^ weeks’ gestation) and with impaired fetal growth ^24^.

The PEA POD, which is designed for use in infants up to 6 months of age or 8 kg in weight, was routinely calibrated and used according to the manufacturer’s instructions in a temperature-controlled room. The newborn baby was evaluated undressed in the test chamber for 2 min and, if necessary, duplicates of irremovable items (clamps, tubes or tags) were measured before the examination.

This ancillary study tests the hypothesis that pregnancy-related etiological factors associated with preterm birth and SGA produce differential body composition proportions that can be evaluated in the neonatal period. It is expected to contribute greatly to the improved phenotypic characterization of these complex syndromes.

### INTERBIO-21 ^st^ Postnatal follow-up Study

At 1 and 2 years of age, anthropometric measurements (weight, length, head circumference, mid-upper arm circumference, triceps skinfold thickness and subscapular skinfold thickness) were taken, and data on the infant’s general health, diet and motor development skills collected.

In addition, neurodevelopment is being assessed at 2 years of age using the INTERGROWTH-21 ^st^ Neurodevelopment Package ^27^. This is a multi-dimensional instrument for early child development ideally suited for both research and screening purposes in field studies and large populations. It was designed to be implemented by non-specialist assessors at individual and small group levels across varied socio-economic and multi-cultural settings. In brief, the Package assesses:

1) Cognition, expressive and receptive language, fine and gross motor skills, behavior, attentional problems and social-emotional reactivity with the INTERGROWTH-21 ^st^ Neurodevelopment Assessment (INTER-NDA). The INTER-NDA consists of 53 directly administered, concurrently observed and maternally reported items rated by trained and standardized assessors. Data are collected via a tablet-based app called the NeuroApp. The INTER-NDA has good reliability across international settings and has been validated against the Bayley Scales ^28^.
2) Cortical auditory evoked response potentials to a novelty oddball paradigm using gel-free, wireless EEG technology (Enobio, Neuroelectrics, Barcelona, Spain) customized for 2 year olds, and software which eliminates the need for specialist training in neurophysiology ^69^. The oddball paradigm consists of a series of frequent, infrequent and novel auditory stimuli, which are presented via wireless earphones to the infant.
3) Sleep-wake patterns and daily physical activity, using a 6-item sleep questionnaire and actigraphy ^70^. The actigraph watch (Motionwatch8, CamNtech Ltd, Cambridge, UK) is worn on the infant’s wrist for 6 days (5 nights) continuously. It measures fine movements at 15 s epochs, and light exposure as well. The data are used to calculate sleep and circadian rhythm parameters, and to quantify daytime physical activity. The operation manuals and protocols are freely available at www.inter-nda.com.
4) Visual acuity and contrast sensitivity with the Cardiff tests, which consist of three sets of 11 grey cards each with a picture at either the top or the bottom of the card ^71^. The cards are presented in a sequence and are used to obtain clinically relevant measures of the child’s visual acuity and contrast sensitivity. Taken together these two observations demonstrate the integrity of the child’s visual pathway.
5) The gross motor domain of the INTER-NDA was complemented with the evaluation of the age of achievement of the matching WHO gross motor development milestones “standing alone” and “walking alone” ^72^. Information was obtained at both the 1 and 2-year follow-up visits in order to evaluate consistency.

These tools were used to assess the eligible 2-year old children in Phase I of the INTERGROWTH-21 ^st^ Project ^29^. Similarities across study sites were measured using variance components analysis and standardised site differences (SSD). In 14 of the 16 domains, the percentage of the total variance explained by between-site differences ranged from 1.3% (cognitive score) to 9.2% (behavior score); 8% or less for the visual and motor items, and <9% for WHO milestones. Of the 80 SSDs comparisons, only six were > ±0.50 units of the pooled SD for the corresponding item. These data demonstrate that the children of healthy, adequately nourished, well-educated pregnant women, recruited from five diverse geographical and cultural study sites, who receive recommended antenatal care, display consistent similarities at 2 years of age across a comprehensive set of neurodevelopmental outcomes. The corresponding normative values have been produced for the evaluation of the INTERBIO-21 ^st^ populations and we would like to continue following these children until at least the age of 5 years.

## Ethics

The INTERBIO-21 ^st^ Study and its ancillary studies were approved by the Oxfordshire Research Ethics Committee “C” (reference: 08/H0606/139), the research ethics committees of the individual participating institutions, as well as the corresponding regional health authorities where the project was implemented. All mothers provided written informed consent for the use of their clinical data and biological samples. Material Transfer Agreements were signed and approved by the relevant national regulatory authorities.

## Discussion

The overall scientific aim of the INTERBIO-21 ^st^ Study is to improve the functional classification of the complex and highly heterogenous preterm birth and FGR syndromes, in particular through a better understanding of the mechanisms that are potentially involved. The aim presupposes that prevention and clinical care could be refined, at both individual and population levels, if the mechanisms were better understood and phenotypes better characterized.

The study directly addresses one of the principal impediments to progress in this field: namely that clinical and public health practice continue to rely upon the use of rudimentary terminology to describe high-risk newborns, e.g. LBW and preterm birth. These old, non-specific definitions, which are based upon arbitrary cut-offs (2500 g and 37 ^+0^ weeks’ gestation) with little differential etiological basis, have probably contributed to the ineffectiveness or unrealistic expectations of interventions that have been used generically in the past, rather than specifically for some of the phenotypes described here. In other words, interventions should no longer be recommended without a detailed investigation of the prevalence of the etiological factors (attributable risks specific for the different phenotypes) at country and regional levels.

An excellent example of the inadequacy of such terms relates to the treatment of malaria in pregnancy. A recent systematic review and meta-analysis of randomized and quasi-randomized trials concluded that prophylactic antimalarial drugs in pregnancy may no longer protect against LBW in areas of high-level antimalarial resistance ^73^. However, only one of the studies included reported SGA as an outcome ^74^; hence, it is unclear whether this lack of apparent protection is a reduced effect on fetal growth or gestational length, or both.

The rationale, therefore, for improving the phenotypic characterization of these complex syndromes in functional terms is simple: the practice of trying to correct the consequences of a sub-optimal intrauterine environment without an accurate assessment of the gestational age at birth and without taking the causes into account makes no biological sense. An accurate clinical and epidemiology-based diagnosis is essential so as to facilitate the appropriate preventive and care regimens, just as it is in other medical specialties.

Over the last decades, it has been repeatedly argued that the use of terms such as LBW was a practical necessity because many countries cannot collect the information required for more detailed characterization, i.e. gestational age estimation. We believe that this approach has had negative effects on three fronts. Firstly, it has not encouraged improvements in data collection in many regions despite technological advances made in diagnostics, imaging, digital health and communications. Secondly, it has perpetuated recommendations of blanket interventions at population level and lastly, it has limited epidemiological and service evaluation efforts. Such a limited approach is increasingly unacceptable for other complex syndromes, i.e. cardiovascular disease ^75^ or malignancy ^76^, even in very resource-deprived regions of the world. Why then should maternal and newborn health be different?

To achieve our proposed paradigm change, we initiated a two-phase research initiative. In Phase I, we produced a set of fully integrated, international standards, based on an accurate estimate of gestational age, that describe optimal growth and neurodevelopment. We used the same prescriptive approach as the WHO MGRS ^44^, by following the babies of a cohort of healthy, educated and well-nourished women from early pregnancy to 2 years of age ^15^. The standards, which perfectly complement the existing WHO Child Growth Standards, should be used to determine the extent to which fetal growth and newborn size deviate from the optimum, particularly at a population level, so as to highlight inequalities in health care and adverse exposures in pregnancy.

In the INTERBIO-21 ^st^ Newborn Case-Control Study, we set out to improve the phenotypic classification of preterm birth and SGA ^11, 12, 77^, using the clinical tools and international standards developed in Phase I, as well as the results of planned nutritional, biochemical, omic and histological studies and other biomarker studies involving healthy and complicated pregnancies, for which the INTERBIO-21 ^st^ Biorepository described above was assembled. 

The strengths of the INTERBIO-21 ^st^ Newborn Case-Control Study are: i) the use of the same training protocols, standardization procedures, data collection methods and quality control measures that were employed in Phase I ^57– 59, 61, 62, 67^; ii) the highly standardized training provided at each site to the dedicated research staff who collected, processed and stored all the biological samples using a protocol that was developed in collaboration with the GAPPS team, and researchers at the Universities of Oxford ^78^ and Cambridge ^79^; iii) the rigorous design of the case-control study, which included the use of a tablet-based app for recruitment, removing the need for the research staff to make any decisions about eligibility, kept them blind to the case or control status of each newborn, and ensured that each participant adhered strictly to the recruitment criteria; iv) the follow-up to the age of 2 years, which includes neurodevelopmental assessment, and v) the availability of newborn body composition data in two of the study sites. 

Collecting biological samples from phenotypically well-characterized cases and controls at the study sites chosen will allow us to explore a wide range of etiological factors and exposures that contribute to the development of complicated pregnancies, which may now seem to present in the same way phenotypically (e.g. low gestational age), as well as the interactions between those risk factors and outcomes.

The overall INTERBIO-21 ^st^ strategy is based on the hypothesis that there are a number of pathways leading to adverse perinatal outcomes that are mediated by multiple molecular, genetic, epigenetic and biochemical mechanisms, with interactive effects from risk factors such as infections, nutritional status and other environmental exposures. The focus on epigenetics arises because of increasing evidence that epigenetic patterns, especially in the placenta, differentially reflect intrauterine exposure to various environmental insults ^80^. It is possible that these inter-related pathways may have differential functional effects at the level of individual fetal organs and physiological systems, with consequences for long-term cardiovascular and metabolic health, and neurodevelopment. 

In summary, the INTERBIO-21 ^st^ Newborn Case-Control Study should provide a unique resource for the planned nutritional, biochemical, omic and histological analyses to enhance current medical knowledge and pave the way for new insights into environmental influences on human growth and neurodevelopment. As the results appear, they will be widely disseminated via traditional routes, e.g. presentations at international meetings and papers in peer-reviewed journals, as well as The Global Health Network ( https://tghn.org) and social media so as to engage the public as much as possible.

## Data availability

No data are associated with this article.
