# Supplementary material for: Deep clinical and biological phenotyping of the preterm birth and small for gestational age syndromes: The INTERBIO-21 st Newborn Case-Control Study protocol
Source: Gates Open Res. 2019 Feb 5;2:49. Originally published 2018 Oct 4. [Version 2] doi: 10.12688/gatesopenres.12869.2 (PMC6545521; doi:10.12688/gatesopenres.12869.2)
Supplement: Peer review history

# Peer review: Reviewer response for version 2

**DOI:** 10.21956/gatesopenres.14021.r27249

**Published:** 2019-05-30

**Authors:** Errol R. Norwitz

**Affiliations:** 1 Mother Infant Research Institute (MIRI), Tufts Medical Center, Boston, MA, USA

Summary

 This paper summarizes the proposed protocol for the INTERBIO-21 ^st^ Newborn Case-Control Study; no original data are presented. This proposal represents a major component of Phase II of the original INTERGROWTH-21 ^st^ project, a worldwide population-based prospective cohort study conducted from 2008-2015 involving nearly 70,000 maternal-infant dyad study subjects and 400 healthcare professionals from 35 institutions in 21 countries. The goal of the original study was to develop a contemporary set of international standards to define normal fetal and childhood growth and neurodevelopment up to 2 years of age, and to compare these standards across different populations throughout the world.

  Phase II—the proposed INTERBIO-21 ^st^ Newborn Case-Control Study outlined in this paper—is intended to leverage the clinical dataset and comprehensive biorepository that has been compiled starting during Phase I and continuing through 2018 at six locations throughout the world (Brazil, Kenya, Pakistan, South Africa, Thailand, and the UK). The goal of Phase II is to better understand the pathophysiology of abnormal pregnancies, specifically those complicated by preterm birth and small-for-gestational age (SGA). Using deep phenotyping of clinical, epidemiologic, and anthropomorphic data from individual maternal-infant dyads and integrating this dataset with linked nutritional, biochemical, multi-omic, and histological information, the investigators aim to better define these heterogeneous syndromes based on a functional (mechanistic) classification. It is likely that such a classification will improve our understanding of how environmental exposures, clinical conditions, and nutrition influence patterns of human growth and neurodevelopment from conception through 2 years of age.

General comments:

 The INTERBIO-21 ^st^ Newborn Case-Control Study addresses an important clinical conundrum—namely, defining the underlying mechanism(s) responsible for the clinical syndromes of preterm birth and SGA—using a unique, research-quality dataset. This paper describes the study population, study design, and methodology that will be used in the study as well as the techniques employed to collect the longitudinal clinical data and biological samples (including maternal blood, umbilical cord blood, placental tissue, maternal feces, and infant buccal swabs). Overall, the study is well conceived and described. Cases were consecutive, and appropriate controls (2:1 ratio) and adjustments have been included. A detailed power analysis is included. A highly respected and experienced team of investigators has been assembled. Standardization of biological sample collections, storage in 1.5mL aliquots, and processing for RNA estimation only if collected within 1 hour (page 10/18, column 2, paragraph 2) represents best practice. Appropriate ethics approval and written patient consent was obtained (page 12/18, column 1, paragraph 3). A few specific comments are listed below.  

Specific comments:

1. Preterm birth and SGA cases included pregnancies delivered at or above 23-0/7 weeks (e.g., page 8/18, column 1, paragraph 3). Why was 23 weeks chosen as the cutoff rather than say 24 weeks, which is more traditional in Europe, or 20 weeks which is commonly used in the US? Please clarify.
2. Similarly, preterm birth is defined in this study as delivery prior to 38 weeks (i.e., up to and including 37-6/7 weeks). This is in contrast to the accepted international definition, which is delivery prior to 37 weeks (i.e., up to and including 36-6/7 weeks). The explanation is included in the text (page 7/18, column 2, last paragraph and page 8/18, column 1, first paragraph) and is based on the observation that neonatal outcome is not as favorable during the 37 ^th^ week (37-0/7 to 37-6/7 weeks) as it is after 38 weeks. This explanation does not make sense. The definition of ‘term’ is just that, a definition; a convention. It is not based on outcome. To follow this argument to its logical conclusion, if it were determined that neonatal outcome is not as favorable during the 38 ^th^ week as it is after 39 weeks, then the definition of preterm birth should be changed to delivery prior to 39 weeks. And if neonatal outcome is not as favorable during the 39 ^th^ week as it is after 40 weeks, then the definition of preterm birth should be changed to delivery prior to 40 weeks. This is not reasonable. The decision to effectively change the definition of ‘term birth’ and ‘preterm birth’ is, in the opinion of this reviewer, a flaw in the study design. Not only will it make it more difficult to extrapolate the findings, but it will create more overlap between the ‘term’ and ‘preterm’ labor phenotypes (i.e., result in less extreme clinical phenotypes) thereby making it less likely that important mechanistic differences will be identified.
3. It is disappointing that no study subjects were included in the post-term period (i.e., delivery after 42 weeks). While it is to late at this time to make any changes, it is the opinion of this reviewer that inclusion of a separate cohort of post-term pregnancies may have provided additional insights into the molecular mechanisms responsible for the onset of labor, both at term and preterm. 
4. There is reference throughout the paper to “nutritional analysis” as an important environmental variable. Does this refer to metabolomic analysis of serum samples? Where other sources of nutritional data included, such as asking study subjects to complete a food diary or a nutritional survey? Will the analysis include detailed data on breast milk vs formula feeding? What about an analysis of breast milk? This would certainly affect growth and neurodevelopment in the first 2 years of life.

I have read this submission. I believe that I have an appropriate level of expertise to confirm that it is of an acceptable scientific standard.

# Peer review: Reviewer response for version 2

**DOI:** 10.21956/gatesopenres.14021.r26909

**Published:** 2019-02-06

**Authors:** Jeffrey S. A. Stringer

**Affiliations:** 1 Division of Global Women’s Health, Department of Obstetrics and Gynecology, University of North Carolina at Chapel Hill, Chapel Hill, NC, USA

No further comments.

I have read this submission. I believe that I have an appropriate level of expertise to confirm that it is of an acceptable scientific standard.

# Peer review: Reviewer response for version 1

**DOI:** 10.21956/gatesopenres.13953.r26696

**Published:** 2018-12-10

**Authors:** Jeffrey S. A. Stringer

**Affiliations:** 1 Division of Global Women’s Health, Department of Obstetrics and Gynecology, University of North Carolina at Chapel Hill, Chapel Hill, NC, USA

Stringer review of Kennedy et al. *Deep clinical and biological phenotyping of the preterm birth and small for gestational age syndromes: The INTERBIO-21 Newborn Case-Control Study protocol.*

 This manuscript provides a detailed description of the study sites, characteristics of target populations, study design / methodology, and procedures for data and sample collection in the INTERBIO-21 ^st ^Newborn Case-Control Study. It is difficult to find much to criticize here. The following notes are minor points that the authors can either take or leave. There is nothing that I found in my review that should delay the indexing of this excellent manuscript. 

- “Deep” is a popular biomedical adjective that some readers may find confusing when applied to clinical phenotyping. The manuscript might benefit from a sentence or two describing what is meant by “deep phenotyping” and how it differs from “phenotyping.”
- Presumably because the study is ongoing, there are instances of confusing verb tense throughout. Some things have been done, others are being done, and still others will be done. Perhaps the copy editors will be able to help with this, but I found it a little distracting as a reader. 
- The section on study design principles gets a bit pedantic (viz, “control selection should be driven by the epidemiological measure of effect that one wishes to estimate”), but ultimately does a good job explaining the rationale for how cases and controls were selected. If the authors wanted to shorten the manuscript, some of this could be replaced with a more straight forward description of what was done with a bit less justification.
- There are 2 instances in the “case-control definitions” section where reference is made to HC as the basis for gestational age determination (“…and whose gestational age was estimated by ultrasound measurement of either crown-rump length <14+0 weeks’ gestation or head circumference <24+0 weeks’ gestation.”  I don’t understand why only HC is mentioned here, when the INTERGROWTH formula uses both HC and FL to estimate GA. Ref 54 is cited, but this is a paper about interobserver variability.
- What are the sample sizes for the ancillary studies (newborn body composition, postnatal follow-up)? Are these ancillary studies being done for all enrollees or only a subset?

 

 Again, a very clearly written paper with very little to quibble over. There is a lot to be learned from this study once results start coming in.

I have read this submission. I believe that I have an appropriate level of expertise to confirm that it is of an acceptable scientific standard.
